# Application and evaluation of the MLVA typing assay for the *Brucella abortus *strains isolated in Korea

**DOI:** 10.1186/1471-2180-9-230

**Published:** 2009-10-29

**Authors:** Moon Her, Sung-Il Kang, Dong-Hee Cho, Yun-Sang Cho, In-Yeong Hwang, Young-Ran Heo, Suk-Chan Jung, Han-Sang Yoo

**Affiliations:** 1 OIE Reference Laboratory for Brucellosis & Zoonosis Laboratory, Bacteriology and Parasitology Division, Veterinary Research Department, National Veterinary Research and Quarantine Service (NVRQS), Anyang, Gyeonggi, Republic of Korea; 2 Department of Food and Nutrition, Chonnam National University, Yongbongdong, Gwangju, Republic of Korea; 3 Department of Infectious Diseases, College of Veterinary Medicine, Brain Korea 21 for Veterinary Science, KRF Zoonotic Disease Priority Research Institute, Seoul National University, Gwanak, Seoul, Republic of Korea

## Abstract

**Background:**

A Brucella eradication program has been executed in Korea. To effectively prevent and control brucellosis, a molecular method for genetic identification and epidemiological trace-back must be established. As part of that, the MLVA typing assay was evaluated and applied to *B. abortus *isolates for analyzing the characteristics of the regional distribution and relationships of foreign isolates.

**Results:**

A total of 177 isolates originating from 105 cattle farms for the period 1996 to 2008 were selected as representatives for the nine provinces of South Korea. A dendrogram of strain relatedness was constructed in accordance with the number of tandem repeat units for 17 loci so that it was possible to trace back in the restricted areas. Even in a farm contaminated by one source, however, the *Brucella *isolates showed an increase or decrease in one TRs copy number at some loci with high DI values. Moreover, those 17 loci was confirmed in stability via *in-vitro *and *in-vivo *passage, and found to be sufficiently stable markers that can readily identify the inoculated strain even if minor changes were detected. In the parsimony analysis with foreign *Brucella *isolates, domestic isolates were clustered distinctively, and located near the Central and Southern American isolates.

**Conclusion:**

The MLVA assay has enough discrimination power in the *Brucella *species level and can be utilized as a tool for the epidemiological trace-back of the *B. abortus *isolates. But it is important to consider that *Brucella *isolates may be capable of undergoing minor changes at some loci in the course of infection or in accordance with the changes of the host.

## Background

Brucellosis is an important disease that is causing economic losses in the cattle industry as well as health problems in humans. Bovine brucellosis in Korea was first detected from cattle in 1955 [1]. Since then, the disease had been occurred sporadically until 1983, and the most outbreaks had been reported in dairy cattle. In spite of the eradication program, the prevalence was continuously increased [2]. For the control and prevention of brucellosis, a new intensive national Brucella eradication program was established and has been executed from July, 2004 in Korea, employing the test-and-slaughter and/or stamp-out approach. All cattle raised in the farms in Korea are regularly tested for brucellosis and a test certificate is required before they could be moved. The brucellosis outbreaks peaked at 2.02% of the tested cattle in 2006 before decreasing gradually to 1.07% in 2007 [2]. In humans, one case of *B. abortus *infection was officially reported in 2002. The number of human cases has continuously increased since then. In 2007, 101 human cases were reported [3].

Brucellosis in cattle is mainly caused by *B. abortus*, which causes herd production losses owing to reproductive problems. *B. abortus *has host preference and infect mainly cattle and other *Bovidae *[4-6]. *B. abortus *has been isolated from a variety of animals, however, among foxes, coyotes, opossums, boars, and raccoons. The infection of dogs and ranched mink by *B. abortus *leads them to undergo abortion, and large numbers of *Brucella *have been cultured from their fetuses and uterine exudates. Vertical transmission has also been reported in coyotes. Some of the *B. abortus *isolates came from the rats in the farms where the cattle were infected, but they do not represent a significant reservoir of brucellosis [4,7-9]. Moreover, *B. abortus *can be transmitted to humans from infected animals through direct contact with the latter's aborted fetuses and fetal membranes, or through the consumption of raw milk and milk products [10,11].

The *Brucella *species have a high DNA homology of greater than 90% [12-15]. The routine identification of the *Brucella *species and biovars has led to their classification through classical biotyping scheme assays using the conventional microbiological tests [16,17]. A few tools have been introduced to molecular genotyping methods, such as polymerase chain reaction-restriction fragment length polymorphisms (PCR-RFLP), random amplified polymorphic DNA (RAPD)-PCR, amplified fragment length polymorphism (AFLP), pulsed field gel electrophoresis (PFGE) and multilocus sequence typing (MLST) [13,18-21]. None of them, however, has proven to be fully satisfactory for epidemiological investigation or for tracing strains back to their origin. The multilocus variable-number tandem repeats (VNTR) analysis (MLVA) methods based on the monitoring of variability in the copy numbers of tandem repeat units (TRs) for several loci were introduced to the assessment of the discrimination potential of genotype-based typing and epidemiological trace-back. TR sequences may be an interesting class of markers as multiple alleles can be presented at a single locus, and as their size differences can be easily resolved through agarose electrophoresis or capillary electrophoresis equipment. MLVA typing based on the number of TRs copy has proven to be a rapid and effective technique for the assessment of pathogenic bacterial species with a high genetic homogeneity, such as *Bacillus anthracis*, *Mycobacterium tuberculosis*, and *Escherichia coli *O157:H7 [22]. It is recently announced that the MLVA typing assay for the *Brucella *species has a good species identification capability and a higher discriminatory power, and that it would thus be proposed as a complement of, or even as a substitute for, the classical biotyping methods [23]. Moreover, this assay shows that it could discriminate the *Brucella *isolates originating from restricted geographic sources, indicating its potential as an epidemiological tool [24-29].

To effectively prevent and control brucellosis in Korea, a molecular method for genetic identification and epidemiological trace-back must be established. As part of that, the MLVA typing assay was evaluated and applied to *B. abortus *isolates for analyzing the characteristics of the regional distribution and relationships of foreign isolates. Moreover, the MLVA loci were confirmed in stability via *in-vitro *and *in-vivo *passages, and the possibility of their use as epidemiological markers for trace-back origin was investigated.

## Results

**Figure 1:**
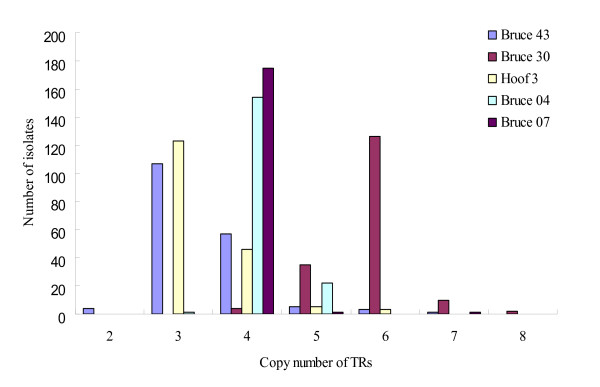
**The 177 prevalent *B. abortus *isolates from five loci appeared to be the allelic type**. The most frequent allelic types in Bruce 04, Bruce 07, Bruce 30, Bruce 43, and Hoof 3, had four, four, six, three, and three copy numbers, respectively.

The tandem repeat units of 17 loci ranged from 6 bp to 134 bp. The PCR products for 17 loci were converted to TRs copy numbers. The PCR product sizes and sequence information usually reflected the exact changes in the number of TRs and were used to predict the TRs copy number in the remaining alleles. Bruce 43, Bruce 30, Hoof 3, Bruce 04, and Bruce 07 for 177 *B. abortus *isolates were detected to have six, five, four, three, and three allelic types, respectively Bruce 43 appeared to have the highest variability. They were shown to have mainly three or four copy numbers of the 12-bp TRs unit, and the rest of the allelic types were shown to have two, five, six, and seven copy numbers. Bruce 30 mainly populated six copy numbers, and Hoof 3 three copy numbers. Moreover, Bruce 04 and 07 had four copy numbers at most (Table 1, Figure 1). The rest of the twelve among 17 loci that were shown to be of a single type were determined to be stable markers for the *B. abortus *isolates in Korea. The DI value was the highest (0.529) at Bruce 43 and was 0.450, 0.448, 0.228, and 0.022 at Bruce 30, Hoof 3, Bruce 04, and Bruce 07, respectively (Table 1).

**Table 1:** Allelic Types and Diversity Index (DI) of 177 *B. abortus *Isolates for 17 loci.

Locus	Allelic types	TRs copy numbers	Diversity index(DI)	Confidence interval
Bruce 04	3	3, 4, 5	0.228	0.153-0.302
Bruce 06	1	4	0	0.000 -- 0.040
Bruce 07	3	4, 5, 7	0.022	0.000 -- 0.053
Bruce 08	1	5	0	0.000 -- 0.040
Bruce 09	1	3	0	0.000 -- 0.040
Bruce 11	1	4	0	0.000 -- 0.040
Bruce 12	1	12	0	0.000 -- 0.040
Bruce 16	1	3	0	0.000 -- 0.040
Bruce 18	1	6	0	0.000 -- 0.040
Bruce 19	1	21	0	0.000 -- 0.040
Bruce 21	1	8	0	0.000 -- 0.040
Bruce 30	5	4, 5, 6, 7, 8	0.450	0.374 -- 0.526
Bruce 42	1	2	0	0.000 -- 0.040
Bruce 43	6	2, 3, 4, 5, 6, 7	0.529	0.476 -- 0.583
Bruce 45	1	3	0	0.000 -- 0.040
Bruce 55	1	3	0	0.000 -- 0.040
Hoof 3	4	3, 4, 5, 6	0.448	0.383 -- 0.514

To detect the changes in each locus for the isolates from farms, two to nine isolates originating from the same farm were selected and a total of 96 isolates from 24 farms were analyzed. The *B. abortus *isolates from 16 farms were found to be of the same type within each farm. Some of the *B. abortus *isolates that originated from eight farms, however, were sometimes found to have two or three allelic types, which had a difference of one copy number for one to three loci (mainly Bruce 30 and/or 43). Moreover, two *B. abortus *isolates in one cow appeared to have a different copy number for Hoof 3 (Table 2).

**Table 2:** A variation of the MLVA profiles for *B. abortus *isolates from the same farms.

Farm	No. of isolates^1)^	No. of isolates for the allelic types^2)^	MLVA profiles^3)^	Comment
CB02	3	3	4-4-4-5-3-4-12-3-6-21-8-5-2-3-3-3-3	

CB03	3	3	4-4-4-5-3-4-12-3-6-21-8-6-2-4-3-3-3	

CN01	6	6	4-4-4-5-3-4-12-3-6-21-8-6-2-3-3-3-3	

GB01	5	4	4-4-4-5-3-4-12-3-6-21-8-6-2-4-3-3-3	
		1	4-4-4-5-3-4-12-3-6-21-8-6-2-**3**-3-3-3	

GB03	9	7	4-4-4-5-3-4-12-3-6-21-8-6-2-4-3-3-3	
		1	4-4-4-5-3-4-12-3-6-21-8-**5**-2-4-3-3-3	
		1	**5**-4-4-5-3-4-12-3-6-21-8-**5**-2-**3**-3-3-3	

GB04	2	1	4-4-4-5-3-4-12-3-6-21-8-6-2-4-3-3-3	
		1	**5**-4-**5**-5-3-4-12-3-6-21-8-6-2-4-3-3-3	

GG01	2	2	4-4-4-5-3-4-12-3-6-21-8-6-2-3-3-3-4	

GG02	3	3	5-4-4-5-3-4-12-3-6-21-8-6-2-3-3-3-3	

GG04	6	6	4-4-4-5-3-4-12-3-6-21-8-5-2-3-3-3-3	

GG05	6	6	5-4-4-5-3-4-12-3-6-21-8-5-2-3-3-3-4	

GG06	3	3	4-4-4-5-3-4-12-3-6-21-8-7-2-4-3-3-3	

GG08	5	3	4-4-4-5-3-4-12-3-6-21-8-7-2-4-3-3-3	
		2	4-4-4-5-3-4-12-3-6-21-8-**8**-2-4-3-3-3	

GG26	3	3	4-4-4-5-3-4-12-3-6-21-8-6-2-3-3-3-3	

GN01	4	4	4-4-4-5-3-4-12-3-6-21-8-6-2-5-3-3-4	

GN02	4	2	4-4-4-5-3-4-12-3-6-21-8-6-2-6-3-3-4	
		1	4-4-4-5-3-4-12-3-6-21-8-6-2-**7**-3-3-4	
		1	4-4-4-5-3-4-12-3-6-21-8-**5**-2-6-3-3-4	

JB01	5	5	4-4-4-5-3-4-12-3-6-21-8-6-2-4-3-3-4	

JJ02	5	3	4-4-4-5-3-4-12-3-6-21-8-6-2-3-3-3-4	
		1	4-4-4-5-3-4-12-3-6-21-8-6-2-**2**-3-3-4	
		1	4-4-4-5-3-4-12-3-6-21-8-6-2-**2**-3-3-**5**	

JN02	3	3	4-4-4-5-3-4-12-3-6-21-8-6-2-4-3-3-4	

JN03	3	3	4-4-4-5-3-4-12-3-6-21-8-6-2-4-3-3-3	

JN05	4	4	4-4-4-5-3-4-12-3-6-21-8-6-2-3-3-3-3	

KW02	3	3	4-4-4-5-3-4-12-3-6-21-8-6-2-3-3-3-3	

KW04^4)^	4	3	4-4-4-5-3-4-12-3-6-21-8-6-2-3-3-3-3	
		1	4-4-4-5-3-4-12-3-6-21-8-6-2-3-3-3-**4**	same cow

KW05	2	2	4-4-4-5-3-4-12-3-6-21-8-6-2-3-3-3-3	

KW08	3	2	4-4-4-5-3-4-12-3-6-21-8-5-2-3-3-3-3	
		1	4-4-4-5-3-4-12-3-6-21-8-5-2-**2**-3-3-3	

^1) ^Majority of the *B. abortus *strains were originated from different cows within the same farm.

^2) ^A number of *B. abortus *strains showing different MLVA profiles were counted.

^3) ^The TRs copy numbers were arranged in the following order loci Bruce 04-06-07-08-09-11-12-16-18-19-21-30-42-43-45-55-Hoof 3. The copy numbers were indicated by bold letters and were underlined.

^4) ^Two strains isolated from one cow were detected to have different allelic types.

**Figure 2:**
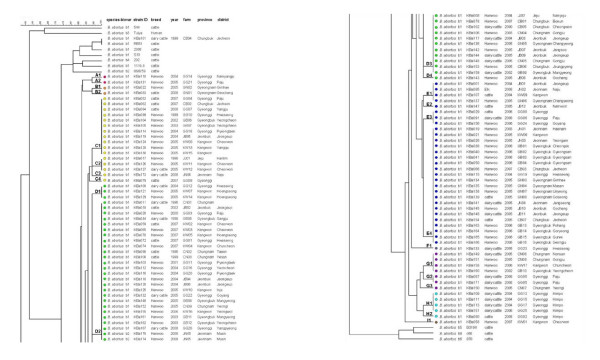
**Cluster analysis for *B. abortus *isolates based on the dataset of 17 loci**. Here was included in 105 *B. abortus *isolates (included RB51 isolate) and 11 *B. abortus *standard strains. All the isolates were confirmed to *B. abortus *strains and were classified into nine clusters and 23 genotypes (A1-I1). In the columns, the following data for isolates were given: species, biovar, strain ID, breed (Hanwoo; Korean native cattle), isolation year, farm, province, and district.

**Figure 3:**
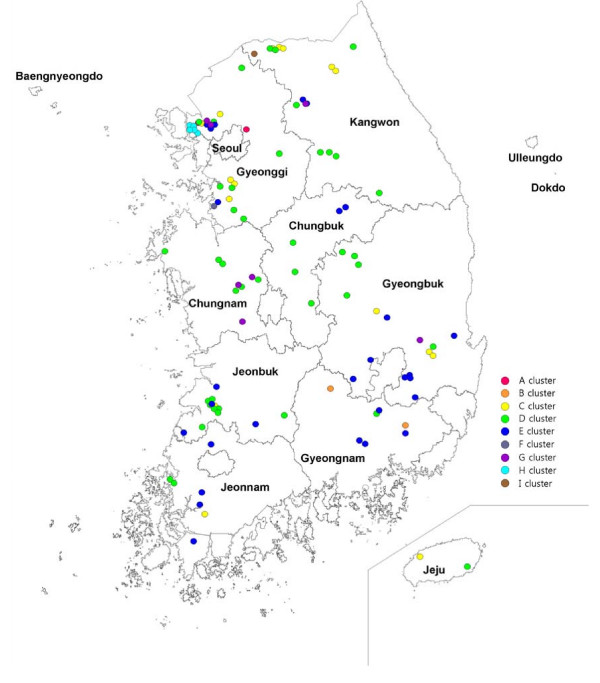
**Geographic distribution of 104 *B. abortus *isolates from Korea**. *B. abortus *isolates were selected in 104 outbreak farms (one strain/farm) from 1996 to 2008.

The *B. abortus *isolates showing a major MLVA profile in a farm were selected to one strain in 104 farms. The dendrogram of strain relatedness was constructed by a character dataset using TRs copy numbers for 17 loci. Through clustering analysis using UPGMA, the *B. abortus *isolates were grouped in clusters showing a 90% similarity. The 104 isolates were classified into nine clusters corresponding to 23 genotypes. The major genotypes were D02, E04, D03, and C01 (Table 3, Figure 2). They have been distributed nationwide and are not closely connected with the provinces. In the local areas or districts, however, the epidemiological connections for the isolates appeared (Figure 2). The isolates with the same MLVA profiles were revealed in the restricted area: in the GB06 and GB07 farms of the C01 genotype in the Gyeonbuk Yeongcheon district; in the KW11 and KW12 farms of the C02 genotype in Kangwon Cheorwon; in the JB02, JB04, and JB06 farms of the D02 genotype in Jeonbuk Jeongeup; in the CB01, CB05, and CB06 farms of the D03 genotype in Chungbuk Boeun, Cheongwon, and Jeungpyeng; and in the GB01, GB02, GB03, GB04, GB13, GB14, GB15, and GB16 farms of the E04 genotype in the Gyeongbuk provinces, among others. These were considered to have been directly or indirectly transferred to the nearby farms. Particularly, the isolates of the H cluster in the Gyeonggi Kimpo area appeared in three neighboring farms in 2004 and were not retrieved. Additional outbreaks occurred in the same area in 2006 and 2008 (Figure 2, 3).

**Table 3:** Distribution of genotypes for 104 *B. abortus *isolates via clustering analysis.

Clusters^1)^	genotypes	MLVA profiles^2)^	No. of isolates^3)^
A	1	4-4-4-5-3-4-12-3-6-21-8-4-2-3-3-3-4	1
	2	4-4-4-5-3-4-12-3-6-21-8-**7**-2-3-3-3-4	1

B	1	4-4-4-5-3-4-12-3-6-21-8-6-2-**6**-3-3-4	1
	2	4-4-4-5-3-4-12-3-6-21-8-6-2-5-3-3-4	1

C	1	4-4-4-5-3-4-12-3-6-21-8-5-2-3-3-3-3	11
	2	4-4-4-5-3-4-12-3-6-21-8-**4**-2-3-3-3-3	3
	3	4-4-4-5-3-4-12-3-6-21-8-**7**-2-3-3-3-3	1
	4	4-4-4-5-3-4-12-3-6-21-8-5-2-**5**-3-3-3	1

D	1	4-4-4-5-3-4-12-3-6-21-8-6-2-3-3-3-**6**	3
	2	4-4-4-5-3-4-12-3-6-21-8-6-2-3-3-3-3	26
	3	4-4-4-5-3-4-12-3-6-21-8-6-2-3-3-3-**4**	11
	4	4-4-4-5-3-4-12-3-6-21-8-6-2-3-3-3-**5**	1

E	1	4-4-4-5-3-4-12-3-6-21-8-6-2-4-3-3-**4**	4
	2	4-4-4-5-3-4-12-3-6-21-8-6-2-4-3-3-**5**	1
	3	4-4-4-5-3-4-12-3-6-21-8-**7**-2-4-3-3-3	3
	4	4-4-4-5-3-4-12-3-6-21-8-6-2-4-3-3-3	21

F	1	4-4-4-5-3-4-12-3-6-21-8-6-2-2-3-3-5	1

G	1	5-4-4-5-3-4-12-3-6-21-8-6-2-3-3-3-4	4
	2	5-4-4-5-3-4-12-3-6-21-8-**5**-2-3-3-3-4	2
	3	5-4-4-5-3-4-12-3-6-21-8-6-2-3-3-3-**5**	1

H	1	5-4-4-5-3-4-12-3-6-21-8-5-2-3-3-3-3	4
	2	5-4-4-5-3-4-12-3-6-21-8-**6**-2-3-3-3-3	1

I	1	5-4-4-5-3-4-12-3-6-21-8-7-2-4-3-3-3	1

Total		9 clusters -- 23 genotypes	104

^1) ^They were grouped according to 90% similarity via clustering analysis, using UPGMA.

^2) ^The TRs copy numbers were arranged in the following order: Bruce 04-06-07-08-09-11-12-16-18-19-21-30-42-43-45-55-Hoof 3. The copy numbers, compared with the major genotypes within a cluster, were indicated by bold letters and were underlined.

^3) ^The *B. abortus *isolates showing a major MLVA profile in a farm were selected (one strain/farm).

Interestingly, an isolate from the CB04 farm in Chungbuk Jecheon in 1999 was confirmed to be *B. abortus *RB51 strain through differential AMOS PCR and the rifampicin resistance test (data not shown). This strain coincided with the MLVA profiles of the standard RB51 vaccine strain, and clustered together. RB51 vaccination was suspended in Korea in 1997, however, as it caused abortions in pregnant cows. This result shows that there is a possibility that the RB51 strain can remain in the body or in a stall for above two years, if not, introduce by unknown mechanism.

**Figure 4:**
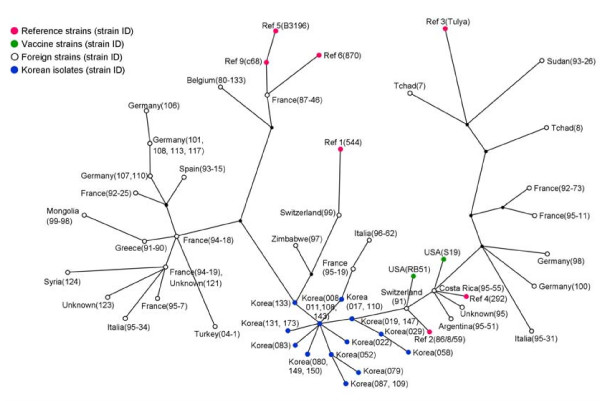
**Maximum parsimony analysis of foreign *B. abortus *strains and Korean isolates**. The data for 48 foreign strains including the reference strain were downloaded from the related websites http://mlva.u-psud.fr[23,30]. There were analyzed by 16 loci, except for Hoof 3, not as information of the foreign strains. The 23 Korean isolates, which were representing 23 genotypes, were compact and were located near the Central and Southern American isolates.

For comparison with the foreign *B. abortus *strains, a dataset of them was downloaded from the related Websites http://mlva.u-psud.fr[23,30]. Forty-eight foreign strains, including the reference strain and 23 *B. abortus *isolates representing the genotypes in Korea, were analyzed by 16 loci, except for Hoof 3, not as information of the foreign strains. In the maximum parsimony analysis with focus on evolutionary modelling, the Korean isolates were compacted and clustered independently. They were located in the middle of the European and African isolates and near the Central and Southern American isolates (Figure 4).

**Figure 5:**
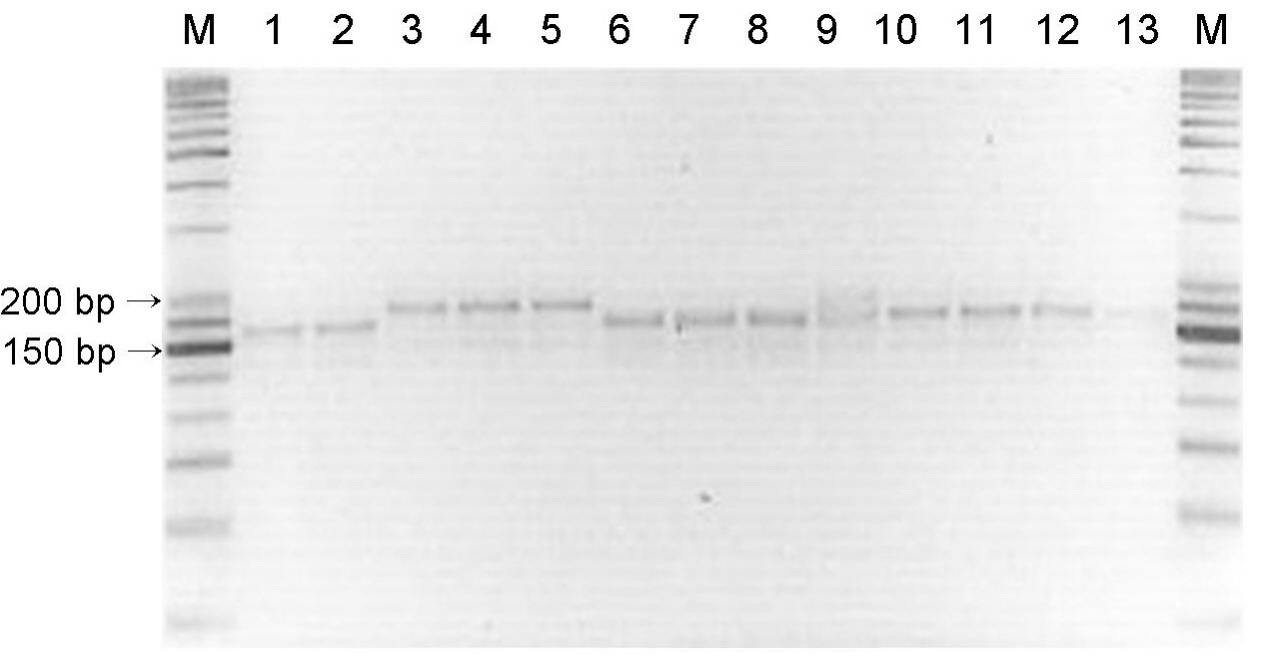
**Variation of the *B. abortus *2308 strains re-isolated via *in-vivo *passage in mice**. Three of the 12 isolates were found to have increased to two TRs copy numbers at Hoof 3. In the rest of 16 loci, no change was detected. M, 25/100 bp ladder; 1, *B. abortus *2308 strain; 2-13, *B. abortus *2308 mouse passage isolates.

To confirm the stability of 17 loci in the same strains, their stability was examined via both the *in-vitro *and *in-vivo *passages. After more than 30 times of *in-vitro *cultivation at two- to three-day intervals, the changes of TRs copy numbers for *B. abortus *544, *B. abortus *2308, and two *B. abortus *isolates were determined. *B. abortus *544 showed an increase in one TRs copy number in the Bruce 04 and 16 at passage 28 times, and a decrease in one TRs copy number in Hoof 3 at passage 29 times (Table 4). But, MLVA profiles for 3 strains except for *B. abortus *544 were unchanged. Also, to measure the stability of 17 loci via *in-vivo *passage, the *B. abortus *RB51 vaccine strains were inoculated in six native Korean cattle and were re-isolated from their lymph nodes. A total of eight isolates were compared with the original *B. abortus *RB51 strain to assess the stability of 17 loci. The MLVA profiles of the re-isolated RB51 strains were identical to that of the original strain, and no change was detected in them, whereas some of the *B. abortus *2308 strains re-isolated via *in-vivo *passage in mouse were shown to have undergone only minor changes at Hoof 3. Three of the 12 isolates were found to have increased two TRs copy number as compared with that of the inoculated *B. abortus *2308 strain. The MLVA profiles for the rest of 16 loci were unchanged (Figure 5).

**Table 4:** Changes of 17 loci during *in vitro *serial passages

Locus	Number of passages that showed a change^1)^				Change of the TRs copy number
		
	*B. abortus* 544	*B. abortus* 2308	*B. abortus* KBa019	*B. abortus* KBa011	
Bruce 04	28	- ^2)^	-	-	An increase in one TRs
Bruce 16	28	-	-	-	An increase in one TRs
Hoof 3	29	-	-	-	An increase in one TRs
14 other loci	-	-	-	-	none

^1) ^Four strains were sub-cultured to fresh media 30 times by serial passages at two- to three-day intervals

^2) ^No change after 30 passages

## Discussion

The six *Brucella *species have been reported to have a high degree of homology (greater than 90%) via DNA-DNA hybridization and their genomes are very similar in sequence, organization, and structure. Moreover, an average amino acid sequence identity was reported to have a high similarity (greater than 99%) [12,13,15]. Due to their high homology in the gene level, the *Brucella *species were only partially differentiated with the use of the molecular genotyping methods based on a number of insertion-deletion events, several polymorphic regions (including the outer-membrane protein-encoding genes), and restriction fragments by enzyme cleavage site. Further, these methods were found not to be fully satisfactory for epidemiologic investigation or for tracing back strains to their origin [13,18-20,31,32].

Recently, a number of bacterial genomes have been fully sequenced. The analysis of the sequenced genomes revealed the presence of variable proportions of repeats, including tandem repeats. Short repeat motifs are known to undergo frequent variation in the number of repeated units [22]. The VNTRs, which are short-sequence tandem repeats, have proven to be a suitable target for assessing genetic polymorphisms within the bacterial species. VNTR-based typing or MLVA has been proven to be an appropriate method for bacterial typing and identification, for determining the genetic diversity, and for the trace-back of highly monomorphological species [22,33,34]. For the *Brucella *species, Hoof-prints, a MLVA assay based on an eight-base pair tandem repeat sequence at eight loci, was introduced as a molecular method for fingerprinting the *Brucella *isolates [24]. Hoof-prints were not appropriate for the discrimination of the *B. abortus *isolates in Korea because of their hypervariability, especially the Hoof 1 and 7 loci, and they need to be replaced by other stable markers [23,35,36]

The MLVA typing assay, designated to some selections of the MLVA loci, was reported to have a good species identification capability and a higher discriminatory power, and could thus be proposed as a complement of, or even as a substitute for, the classical biotyping methods [23,27,30]. This assay showed that it could discriminate isolates originating from restricted geographic sources, indicating its potential as an epidemiological tool [25-27]. Genetic diversity of the *Brucella *isolates must be investigated, and the epidemiological trace-back tool must be evaluated, for the effective prevention of brucellosis. Thus, we endeavoured to assess the MLVA typing assay of the *B. abortus *strains isolated in Korea based on 17 primer sets, which were consisted of 16 markers described previously [23,30] and Hoof 3 used by hoof-prints [24]. Hoof 3 was able to differentiate the *B. abortus *RB51 vaccine strain (TRs copy number: 4) from its mother strain, *B. abortus *2308 (TRs copy number: 5), and was shown to have the discrimnation power of a moderate stable marker (Table 1). As it caused abortion in pregnant cattle, *Brucella *RB51 vaccination was suspended in Korea in 1997. In late 1999, however, one *B. abortus *strain isolated from dairy cattle was identified as the RB51 vaccine strain using the classical biotyping scheme and differential AMOS PCR [17,37], and its strain was confirmed to completely coincide with the original strain by 17 loci, especially Hoof 3 (Figure 2). This result shows that Hoof 3 can be increased the discrimination capacity and trace-back ability of the MLVA assay.

The 177 strains isolated from 105 cattle farms in nine provinces in Korea from 1996 to 2008 were investigated in this study [see additional file 1]. Bruce 43 appeared to have a variety of alleles, and its DI value was the highest at 0.529 (Table 1). In addition, the *B. abortus *isolates that originated from the same farms at the same time were sometimes found to have a difference of one copy number for mainly Bruce 30 or 43 (Table 2). Le Fleche et al. [23] divided the 15 loci into two groups, one consisting of eight loci with a good species identification capability (panel 1) and another complementary group of seven loci with a high discriminatory power (panel 2). Bruce 43 was included in panel 1 and was reported to be a moderately variable marker. Moreover, Al Dahouk et al. [30] reported that Bruce 43 had three alleles and a 0.22 DI for the 43 *B. abortus *strains isolated from animals (except for a single human isolate). The results of this study show, however, that Bruce 43 is a higly variable marker with six alleles and 0.529 DI, and that it is sometimes found to have a different copy number in the same farm (Table 1, 3). Therefore, Bruce 43 needs to serve as a rather discriminating marker than as a species identification marker for the *B. abortus *strains. Bruce 30 (Hoof 2), however, was found to have five alleles and a 0.450 DI, which is slightly lower than five alleles as well as a 0.69 [30] and a 0.72 DI [27]. Hoof-3 and Bruce 04 (Hoof 6) were found to have 0.448 and 0.228 DIs, lower than the 0.83 and 0.68 DIs [27] or 0.630 and 0.535 DIs [36] previously reported. Moreover, the DI values at the other loci, except for Bruce 43, Bruce 30, Hoof-3, and Bruce 04, range from 0 to 0.022 (Table 1), which are very much lower than the 0-0.75 DIs reported in the 43 *B. abortus *isolates previously [27,30]. These low DI values are as expected if the population of *B. abortus *isolates present in Korea was the result localized by clonal expansion of *B. abortus *strain without the input of a new strain recently.

To detect the changes in the MLVA profiles for the isolates within the same farms, a total of 96 isolates from 24 farms were analyzed. Some of the *B. abortus *isolates that originated from seven farms were found to have two or three allelic profiles in the same farm, with a difference of one copy number for Bruce 30, Bruce 43, or Hoof-3. Particularly, two *B. abortus *isolates that originated from one cow in the KW04 farm appeared to have one copy number difference in Hoof-3 (Table 2). In the results of the epidemiological investigation, each of the seven farms did not seem to have mixed infections from the strains that originated from different sources. In the course of replication in the body, emission to an environmental material by abortion, resistance of any external condition, and re-infection during their existence within a stall, mutants can be generated at the genetic sites that code TRs. Whatmore et al. [27] reported, after the experimental infection of pigs with *B. suis*, that the strains that were re-isolated from four of six infected animals showed some minor changes, an increase or decrease in one TRs copy number. They were identified to have mutation events at four loci, showing a high DI within the *B. suis *strains. In general, random genetic events, including the insertions, deletions, and point mutations of DNA, have been generated commonly in the course of an outbreak [38]. The *Brucella *species are not exceptions to these genetic events. It was reported that erythritol-tolerant mutants generated a proportion ranging from 10^-4 ^to 10^-6 ^in the *B. abortus *S19 vaccine strain [39]. Changes in the TRs copy number of each locus are possible, and there are generally different mutant rates at different genetic sites [40]. An isolate is mostly considered to be closely related to the outbreak strain if its PFGE pattern differs from the outbreak pattern based on the changes consistent with a single genetic event, which result in two to three band differences [38]. Similarly, isolates that change to one copy number for one to two loci in the same farm and at the same time, especially loci that have high DI values, will have to be regarded as strains that originated from the same source, or as closely related strains. Thus, a cluster was classified into a group showing a 90% similarity via clustering analysis, with a difference of only one to three copy numbers (Table 3).

Clustering analysis was performed with major isolates selected from 104 farms. They were classified into nine clusters and 23 genotypes. The major genotypes have been distributed nationwide and their geographic characteristics have not been found. In the local areas or districts, however, genetic horizontal transfers, which are epidemiological connections for farm to farm, were detected in a majority of genotypes. Moreover, some clusters (for example, the H cluster) were indicated to be circulating in a specific local area, and were continuously confirmed to re-infect the neighboring farms by year (Figure 3). The MLVA profile analysis that was conducted on the basis of the TRs copy numbers of 17 loci showed potentiality as an epidemiological tool in the restricted area. Its use as an epidemiological tool with the MLVA assay has already been reported [26,41]. For 24 *B. melitensis *human isolates, the MLVA assay appeared to assist with the investigation of outbreaks. The isolates that clustered together in the same MLVA genotype indicated a common source of infection. According to the results of MLVA assay, a laboratory technician was proved to have an infection in the laboratory. Clinical, environmental, and animal isolates through the MLVA assay could allow the testing of the hypotheses regarding outbreak confirmation, extent of transmission, source, and reservoir. This assay encourages the use of a molecular method in epidemiological trace-back analysis.

The maximum parsimony analysis of 48 foreign *B. abortus *strains and 23 Korean *B. abortus *isolates was performed. The Korean isolates were not highly divided and were compact. When comparing with database (Brucella 2007) on the website http://mlva.u-psud.fr[23,30], the Korean isolates profiles were similar to the genotype 27 or 28 in panel 1, but they represented new genotypes. They were located near the Central and Southern American isolates (Figure 4). These results seem to prove that the *B. abortus *isolates have been localized by clonal expansion without the influx of other new strains, by the strict national quarantine.

The stability of 17 loci was examined via both the *in-vitro *and *in-vivo *passages. In the *in-vitro *passage, *B. abortus *544 showed only an increase or decrease in one TRs copy number at Bruce 04, 16 and Hoof 3 toward the end of passage course (Table 4). Whatmore et al. reported, after three strains, the *B. suis, B. melitensis*, and *B. abortus *isolates were passaged *in vitro *14 times over 270 days, that only the *B. abortus *isolate showed an increase in one TRs copy number at one locus (VNTR 12B) towards the end of this time course [27]. This locus that showed a change was hypervariable to DI 0.88. The clinical isolates would, however, prior to routine, undergo the MLVA assay, which indicates that *in-vitro *cultivation will not lead to significant changes in the MLVA profiles [27]. To measure the stability of 17 loci via *in-vivo *passage, native Korean cattle and ICR mice were experimentally infected with the *B. abortus *strains. The *B. abortus *RB51 vaccine strains inoculated in the Korean native cattle were not found to have undergone any change in 17 loci, but some of the *B. abortus *2308 strains that were isolated in the mice were found to have increased TRs copy numbers at Hoof-3 (Figure 5). Although this difference was naturally caused, it may be generated in the course of the adaptation to the changes in the host. If brucella isolates are transferred to the non-preference hosts, there may be changed to TRs copy numbers in some of 17 loci. As the *B. abortus *strain has infected various animals besides the *Bovidae*, there seems to be a need for these changes to be further investigated in using the MLVA assay as an epidemiological trace-back tool for transmissions between natural and heterogeneous hosts.

## Conclusion

Korean *B. abortus *isolates were clustered into nine clusters and 23 genotypes, although they were not highly divided and had low DI values. The MLVA assay showed enough discrimination power in the *Brucella *species level and could thus be utilized as a tool for epidemiological trace-back in a restricted area. Moreover, it must be considered that even in the farm that was contaminated by one source, the *Brucella *isolates were able to undergo minor changes at some loci with high DI values especially. The stability studies performed via the *in-vivo *and *in-vitro *passages showed that although further investigation may be needed to determine the stability of marker by changes of the host, 17 loci in this study are sufficiently stable markers for the identification of the original inoculation strain. The MLVA assay can also be applied to determine the relationship between the *Brucella *isolates from animals and from humans.

## Methods

### *B. abortus *isolates and DNA template preparation

A total of 177 isolate that originated from 105 cattle farms (including one elk farm) for the period 1996 to 2008 were selected as representatives for the nine provinces of Korea, namely: Chungbuk (CB), Chungnam (CN), Gyeongbuk (GB), Gyeongnam (GN), Gyeonggi (GG), Jeonbuk (JB), Jeonnam (JN), Jeju (JJ), and Kangwon (KW) [see Additional file 1]. They were all identified using AMOS PCR and the classical biotyping scheme of the *Brucella *species based on Gram's staining, oxidase and catalase production, urease activity, H_2_S production, CO_2 _requirement, growth in the presence of basic fuschin and thionine (20 μg/ml), agglutination with monospecific sera, and lysis by phages [16,17,37,42]. Most of them were confirmed to be *B. abortus *biotype 1, and eight strains that were isolated two times from a farm were found to be *B. abortus *biotype 2.

The *B. abortus *isolates were cultured on a tryptic soy agar supplemented with 5% bovine serum for three to five days at 37°C, under 5% CO_2_. The genomic DNA of the isolates was extracted using a DNeasy blood & tissue kit (Qiagen Korea Ltd., Korea), according to the manufacturer's instructions, and was stored at -20°C until further use.

### Seventeen MLVA loci and TRs copy number verification

Seventeen loci for the MLVA typing assay were consisted of the primer sets of 16 loci described by Al Dahouk et al. [23,30] and Hoof 3 described by Bricker et al. [24]. The forward primer of each primer set was synthesized with one of three fluorescent dyes (HEX; green or 6-FAM; blue) covalently bound to the 5'-end of the primer. PCR amplification was performed using *AccuPower *PCR premix (Bioneer Co, Korea). The PCR conditions were as previously described [23]. Amplification was performed using a T3000 Thermocycler (Biometra, Germany). The PCR product sizes of all the loci were ascertained with the use of a 25/100-bp DNA ladder via 3%-agarose-gel electrophoresis and were compared with the internal standard strains (*B. abortus *biovar 1, 544 and biovar 4, 292 referencestrains). Moreover, to obtain their correct sizes for the locus showing alleles, the PCR products were purified by passing them through a QiaQuick PCR purification column (Qiagen), and were diluted between 1:10 to 1:100 in distilled water, depending on the estimated concentration. A 1-ul aliquot was fit into an Applied Biosystems 3730xl DNA Analyzer (USA) with filter set G5. A GeneScan LIZ^®^500 size marker (Applied Biosystems) as an internal standard, and the bands were sized relative to these markers by using the GeneMapper^® ^software ver. 3.7 (Applied Biosystems).

### Genetic diversity

The genetic diversity of the isolates was determined using Simpson's diversity index (DI). The DI was calculated using the V-DICE (VNTR diversity and confidence extractor) program in the HPA-Bioinformatics online tools http://www.hpa.org.uk. The DI is a measure of the variability of the TRs copy number at each locus. It can range from zero (no diversity) to one (extreme diversity). A locus whose samples have similar TRs copy numbers will have a lower DI value, whereas a locus whose samples almost all have different TRs copy numbers will have a very high DI value. Moreover, the confidence interval (CI) generated for each examined locus indicates the precision of the DI by providing the upper and lower boundaries.

### Data analysis for 17 loci

The TRs copy numbers for the 17 loci of the isolates were inputted into a character dataset using Bioumerics ver. 5.1 (Core-Bio, Korea). In addition, all the general information regarding the isolates was recorded by year of isolation, farm, province, address, etc. Clustering analysis was performed using UPGMA (unweighted pair group method using arithmetic averages) with the categorical similarity coefficient, and the maximum parsimony was analyzed.

### Stability of 17 loci via *in-vitro *and *in-vivo *passage

To determine the stability of each locus via *in-vitro *passage, *B. abortus *544, *B. abortus *2308, and two *B. abortus *isolates were inoculated on a 20-ml tryptic soy broth supplemented with 5% bovine serum at 37°C, under 5% CO_2_, and were sub-cultured to fresh media 30 times, by serial passages, at two- to three-day intervals. The DNA of the strains cultivated in each passage was extracted and was subjected to MLVA analysis.

For the *in-vivo *experiments, six approximately eight-month-old Korean native cattle (Hanwoo) were vaccinated with one dose of the *B. abortus *RB51 vaccine (Colorado Serum Company, USA). Four weeks after the inoculation, two cows were slaughtered at two-week intervals, and vaccine strains were re-isolated from their lymph nodes. The isolated strains were confirmed using AMOS PCR and the classical biotyping scheme. The eight re-isolated strains were compared with the original strain to assess the stability of 17 loci. Moreover, the *B. abortus *2308 strains were inoculated in six mice via the intraperitoneal route. They were re-isolated from each spleen of dead mouse after two to three days. Two strains from each mouse were randomly selected onto 5% sheep blood plate. The 12 recovered strains were tested to assess the stability of 17 loci based on the changes in the host. (This experiment has been approved to animal experiment ethical committee of NVRQS. Approval number is NVRQS-AEC-2008-12)

## Authors' contributions

MH designed the study, carried out strain selection and biotyping, analyzed the data related to strain relatedness and clustering analysis, and also drafted the manuscript. SIK was in charge of DNAs preparation, agarose-gel electrophoresis and PCR product analysis. DHC, YSC and IYH carried out animal examination, and checked data related strain information. YRH helped to execute Bioumerics program and to analyze the MLVA data. SCJ and HSY provided intellectual input, and helped to draft the manuscript. All authors read, commented, and approved the final the manuscript.

## Supplementary Material

Additional file 1 **Dataset of *B. abortus *strains used in this study**. The data provided the strains information, their genotypes and MLVA data of 17 loci. Click here for file
